# Effectiveness of Puncture-Aspiration-Injection-Reaspiration in the Treatment of Hepatic Hydatid Cysts

**DOI:** 10.5812/iranjradiol.7370

**Published:** 2013-05-20

**Authors:** Raman Rajesh, Dhiman S. Dalip, Jhobta Anupam, Azad Jaisiram

**Affiliations:** 1 Department of Radiodiagnosis, JSS Medical College, Mysore, India; 2 Department of Radiodiagnosis, Indira Gandhi Medical College, Shimla, India

**Keywords:** Echinococcosis, Image-Guided Biopsy, Povidone Iodine

## Abstract

**Background:**

Hydatid disease of the liver is endemic in cattle rearing areas of the world. A variety of treatment options are available in its management. The common treatment options are medical therapy, surgery and puncture-aspiration-injection-reaspiration (PAIR) therapy.

**Objectives:**

This study was performed to evaluate the effectiveness of PAIR therapy in the treatment of hepatic hydatid disease.

**Patients and Methods:**

This cross sectional study was carried out on 15 consecutive patients (Male: 2, Female: 13; Age group: 11-80 years) with hepatic hydatid disease and were treated by PAIR therapy and followed up for a period of 1 year. The cysts were punctured under local anesthesia with an 18G needle using sonographic guidance. Betadine (10% povidone iodine + 1% free iodine) was used as scolicidal agent and allowed to act for 30 min. Cysts larger than 5 cm (n = 5) were drained using an 8F pig tail catheter. The therapeutic response was studied by assessing the reduction in the cyst size, progressive solidification of the cyst, calcification of the wall and increase in the echogenicity of the cyst with pseudomass appearance on serial ultrasound examinations performed on the next day, after 1 month, at 3 months, 6 months and 1 year after the procedure.

**Results:**

Ten patients (66.7%) had Gharbi type I cysts, two (13.3%) had type II and three (20%) had type III cysts. All the patients (100%) showed reduction in cyst size over a 3-6 month period. Pseudomass appearance with solidification was seen in 73% of the patients and calcification was seen in 46.6%. None of the patients developed anaphylaxis, recurrence or peritoneal seedlings. Pain at the injection site was the most common complication observed.

**Conclusion:**

PAIR therapy is an effective minimally invasive treatment for Gharbi type I-III hepatic hydatid cysts. It is a cost effective and safe procedure with significant reduction in the duration of hospital stay.

## 1. Background

Cystic Echinococcosis or hydatidosis is an infestation caused by larval forms of the dog tapeworm, Echinococcus granulosus. It is endemic in the Mediterranean region, South America, Australia, New Zealand and cattle rearing areas like Turkey and South East Asia (1). Hydatid cyst can develop in any organ of the body, but commonly occurs in the liver. The right lobe of the liver is affected more frequently than the left (2). The patients are usually asymptomatic and the cyst is incidentally detected on ultrasonography performed for some other purpose (3). The clinical features depend on the site, size of the cyst, pressure effects on adjacent structures or rupture (4). Clinical symptoms and signs include vague pain in the upper abdomen, fullness of the abdomen, jaundice whenever there is biliary communication, abdominal distention and ascites when the cyst ruptures. In the past, surgery was considered as the only effective treatment of hydatid disease (5). In the early 1970s, benzimidazole carbamates like mebendazole and albendazole proved to be effective against larvae of E. granulosus. In the 1980s, reports of accidental punctures of hydatid cysts without any complications contributed to deliberate puncture of hydatid cyst followed by the introduction of a scolicidal agent (6). This method was known as puncture-aspiration-injection-reaspiration (PAIR) and was recommended by the WHO as an alternative method to surgery (7). Recently, minimally invasive treatments like laparoscopic surgery and PAIR therapy are becoming popular for the treatment of hydatid cyst (2, 4, 8). Percutaneous treatment of hydatid cyst of the liver was first reported by Mueller et al. in 1985 (9). Since then, many studies have placed PAIR therapy as an alternative treatment to surgery. Serious complications such as anaphylactic shock or death did not occur in these studies. Long-term results indicate that percutaneous treatment of liver hydatid cysts is an effective treatment for type I, II and III cysts (3, 6, 10, 11) that can be used liberally in these cases.

## 2. Objectives

The objective of this study was to evaluate the effectiveness of PAIR therapy in the treatment of hepatic hydatid cysts.

## 3. Patients and Methods

### 3.1. Patient Selection

This study was carried out in the department of radiodiagnosis, Indira Gandhi Medical College, Shimla, India from April 2007 to April 2009. No grants were obtained from any support source for the study. Inclusion criteria were patients having single/multiple hepatic hydatid cysts, adequate liver parenchyma (>5 mm) surrounding the cyst, cyst size >3 cm and patients who refused surgery or had relapse after surgery. Exclusion criteria were cysts which were inaccessible to puncture, peripheral cysts having <5 mm of surrounding hepatic tissue, cysts with communication to the biliary tree, history of anaphylaxis or atopy, uncooperative patients and patients with Gharbi type IV and V cysts. Written informed consent was obtained from all the patients before the procedure. The study was approved by the ethical committee of the hospital.

### 3.2. Procedure

PAIR therapy was performed on 15 patients with hepatic hydatid cysts. After detailed history and clinical examination, the diagnosis was established on sonography and serological test by ELISA (12). The cysts were classified according to Gharbi classification (13). According to this classification, type-I hydatid cyst refers to a simple cyst without septae, floating membranes and daughter cysts, type-II cyst refers to a cyst with floating membranes; type-III cyst is a hydatid cyst with daughter cysts, type-IV cyst is a cyst with internal echoes and solid areas and type-V cyst refers to areas of calcification in the cysts. CT scan was only performed when calcification in the cysts and relation to the vessels and biliary tree needed to be established. The measurements of the cyst size and the surrounding liver parenchyma were performed using ultrasonography. Albendazole tablet (400 mg or 10 mg/kg body weight twice daily) was started four days prior to the procedure.Emergency tray consisting of drugs like atropine, adrenaline, hydrocortisone and chlorpheniramine maleate was ready before the procedure. The procedure was performed by sonographic guidance (GE RT 3200 machine), under local anesthesia. Initial puncture was performed by the free hand technique using an 18G needle, through transhepatic approach (Figure 1). The entire fluid in the cyst was aspirated leaving behind only just little fluid to allow visibility of the needle tip (Figure 2). The aspirated hydatid fluid was tested by dip stick (Multistix SG; BAYER Diagnostics) test (Figure 3) for the presence of bile salts and pigments to rule out biliary communication.

**Figure:**
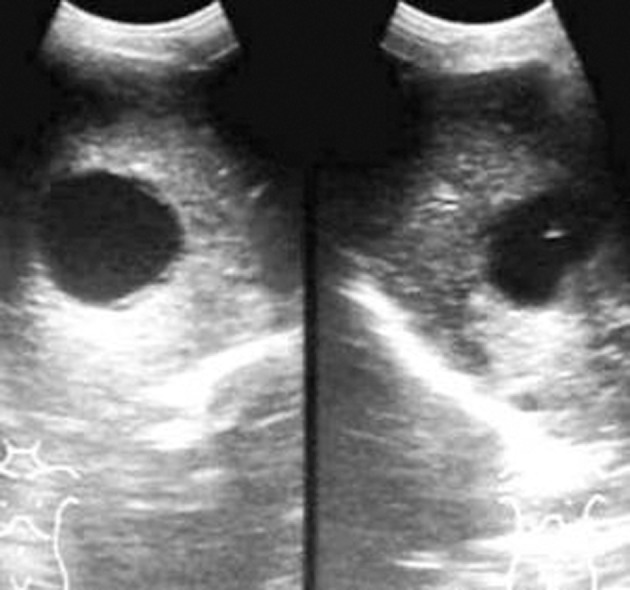
Figure 1. Control image of Gharbi type-I hepatic hydatid cyst on the left side and needle tip in the cyst on the right side (puncture)

**Figure:**
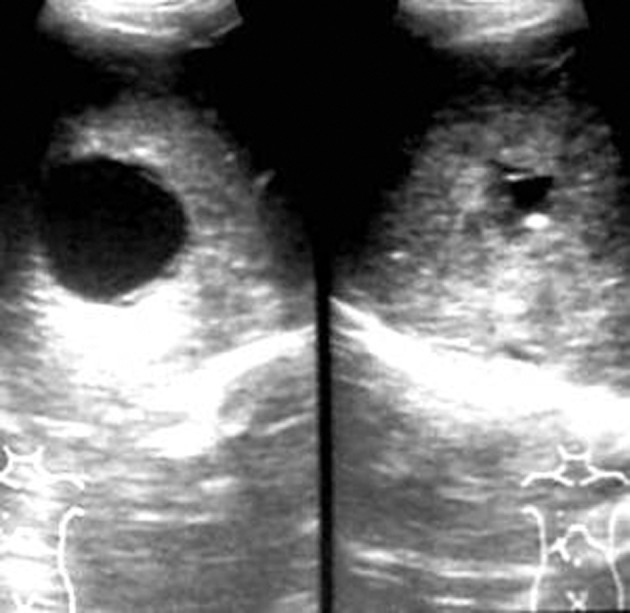
Figure 2. Aspiration of the cyst

**Figure:**
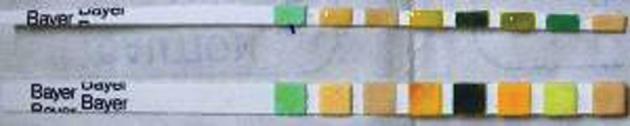
Figure 3. Test strip used for the detection of bile salts and bile pigments in the cyst fluid (Multistix SG; BAYER Diagnostics)

CT scan was performed to evaluate the biliary communication in cases with a positive dip stick test. After confirmation of the absence of biliary communication, the scolicidal agent–betadine (10% povidone iodine, 1% free iodine)–was injected. In cysts smaller than 5 cm, almost complete aspiration of the cyst was performed, followed by instillation of the scolicidal agent (Figure 4) into the cyst cavity which was left in situ for 30 min. The volume of the scolicidal agent was two thirds of the aspirated hydatid fluid volume or a maximum of 50 ml. The stylet was reintroduced during the acting period of 30 min. The contents were reaspirated after 30 min (Figure 5).

**Figure:**
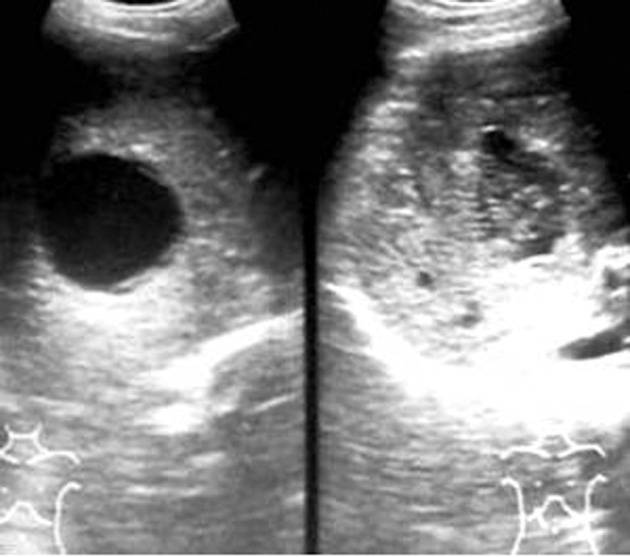
Figure 4. Injection of scolicidal producing internal echoes in the cyst

**Figure:**
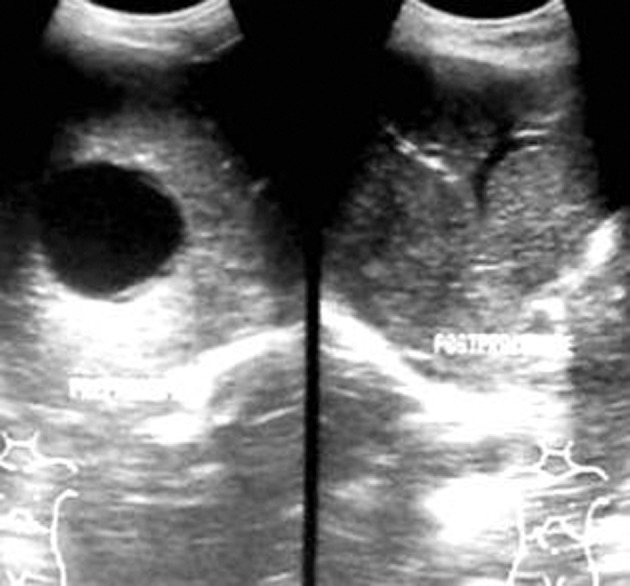
Figure 5. Reaspiration of the cyst

In cysts larger than 5cm, catheter drainage was performed. In such cases, after needle puncture and test aspiration, the tract was dilated over a compatible guide wire using dilators. An 8F pig tail catheter was introduced and rest of the cyst contents were aspirated through the catheter. The scolicidal agent was injected and the catheter was clamped for 30 min, after which the injected scolicidal was reaspirated. A second dose of the same quantity of scolicidal was injected and allowed to drain into the drainage bag without aspiration. The catheter was removed after the drainage stopped or was less than 20 ml per day. When membranes clogged the needle/catheter and prevented aspiration, stylet/guide wire was reintroduced to flush the membranes. Vital signs were monitored throughout the procedure. The catheter was removed when the drainage ceased completely or was less than 20 ml/day. Follow-up ultrasonography was performed on the next day after the procedure, at one month, three months, six months and one year. Oral albendazole 400 mg BD was given for one month and was continued for three months in case of low response at one month follow-up sonography.

### 3.3. Criteria of Good Response

Criteria of good response were defined as reduction in the size of the cyst, changes in the echo pattern with appearance of solid areas in the cyst, progressive solidification of the cyst, calcification of the wall, and increase in echogenicity of the cyst with pseudomass appearance.

## 4. Results

### 4.1. Presenting Symptoms

Most of the patients presented with vague upper abdominal pain (n=8; 53.3%). In four patients (26.7%), the predominant complaint was abdominal discomfort. Two patients (13.3%) had breathlessness. One patient, who also had a lung hydatid cyst, complained of both right upper abdominal pain and hemoptysis (6.7%). After PAIR therapy, there was resolution of the abdominal pain. However, the upper abdominal discomfort persisted in two (13.3%) patients. This was probably due to the large initial size of the cysts prior to therapy.

### 4.2. Size and Shape of the Treated Cysts

Two hydatid cysts were oval in shape (13.3%) and the remaining 13 cysts (86.7%) were round. The size of the cysts ranged from 4 × 3 cm to 15 × 7 cm with an average of 9.5 × 5 cm. Eight (53.3%) of the cysts were less than or equal to 5 cm in diameter. The remainder of the cysts were larger than 5 cm in diameter (n=7; 46.7%). After PAIR therapy, the average size of the cysts was about 3.5 × 2.0 cm at three-month follow-up sonography suggesting an approximate 70% reduction in cyst size.

### 4.3. Number and Location of the Cysts

In the present study, out of 15 patients, 10 patients had single hydatid cyst (Figure 1). Two patients had two cysts, and three patients had three cysts. In patients with multiple cysts, the largest cyst was treated first, followed by smaller cysts in further sittings. Four patients had cyst in segment IV and VI; five in segment II, one each in segment V and VIII. One patient had cyst extending into both segments VI and VII. No patient had hydatid cyst in segment I. The majority of the cysts were Gharbi type I (n=10; 66.7%). Two cysts were Gharbi type II (13.3%). Three patients had type III cysts (20%). One patient had type III as well as type IV cysts. In this case, only type III cyst was treated. Five cysts (33.3%) were drained with 8F pigtail catheter using Seldinger technique. The remaining ten cysts (66.7%) were treated by simple PAIR therapy alone.

### 4.4. Duration of the Procedure

The duration of the procedure ranged from 35-60 min (mean, 47.5 min). The procedure was completed in less than or equal to 40 min in seven patients and it took more than 40 min in eight patients.

### 4.5. Complications of the Procedure

Pain at the injection site was the most common untoward incident seen in nine patients (60%). Urticaria and leakage were each noted in one patient (6.7%). Serious complications like anaphylaxis and life threatening reactions were not observed in any of the patients. One patient had peritoneal leakage. This patient had a surrounding liver parenchymal thickness of 5 mm. On follow-up sonography, 150 ml of the free fluid was present in the pelvis. This patient was treated with intravenous gentamicin and ceftriaxone and kept under observation. On repeat sonography on the fourth day, there was minimal free fluid in the pelvis. It resolved over a week after conservative treatment. On the subsequent follow-up ultrasonography, no peritoneal seedling was noted. Secondary seedling or recurrences were not seen in any patient during the one-year follow-up period. Serious complications such as anaphylaxis and death were not seen in any cases. Six of the patients (40%) had no complications.

### 4.6. Response to Therapy

All the patients showed good response to PAIR therapy. On the 3-month follow-up sonography, the the size of cyst was reduced in all 15 patients, solidification/pseudotumour was seen in 11 (Figure 6), foci of calcification in seven and residual cyst in four of the patients.

**Figure:**
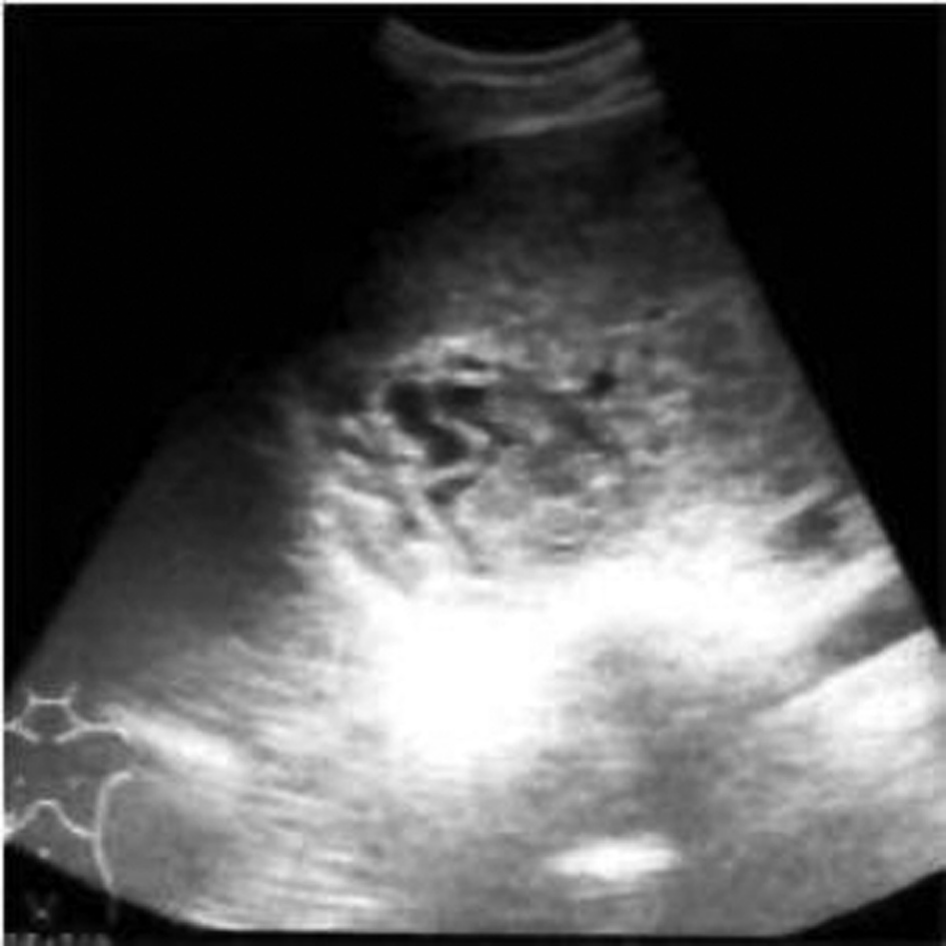
Figure 6. Follow up sonography at three months showing pseudotumor appearance

### 4.7. Duration of Hospital Stay

The duration of hospital stay ranged from 2 to 14 days with an average of four days. The patient with 14 days of hospital stay had co-morbid medical problems.

## 5. Discussion

Hydatid disease is an infestation caused by Echinococcus granulosus larvae, in which man is an accidental intermediate host. The liver acts as the first filter in the disease and lungs act as the second filters (14). There are various treatment modalities for this disease. The most common and well-known treatment is surgery. However, surgery is associated with significant morbidity and long hospital stay. Minimally invasive methods of treatment such as laparoscopic surgery and percutaneous therapy have the advantage of less morbidity, low cost, and shorter hospital stay. PAIR therapy along with oral anthelmintic therapy is very efficacious and results in better outcome than surgery in Gharbi type I-III cysts (13). In our study, only Gharbi type I-III hydatid cysts were treated. Type IV cysts contain echogenic material which is tough to drain and type V cysts show calcification which is a sign of healing and natural evolution of the hydatid cyst, hence excluded from the study. Bosanac et al. (15) performed the initial puncture using 18G needle, through transhepatic route, under sonographic guidance. In our study, the cysts were also punctured transhepatically using an 18G needle under sonographic guidance and local anesthesia. The transhepatic route was chosen as it provides adequate surrounding liver parenchyma, which prevents leakage and gives good tamponade effect for the cyst cavity during catheter drainage. In two cases, the daughter cysts appeared from within after initial puncture and aspiration. These cysts were invisible during routine sonographic examination. The daughter cysts were also punctured using the same needle. Saremi et al. (16, 17) used a new cutting device to cut the membranes of the hydatid cyst. No cutting device was used in the present study. The collapsed membranes remained in the cyst cavity and gradually solidified. If the membranes clogged the needle/catheter lumen, then stylet/guide wire was inserted to get rid of the membranes from the needle/catheter. Many scolicidal agents have been used by various investigators in their studies. Ninety five percent alcohol was used in a few studies (15-17), 20% hypertonic saline was used by some investigators (1, 18); albendazole was also used in a few studies (19). Bosanac et al. (15) used betadine (10% povidone iodine; 1% free iodine) as the scolicidal agent for 30 min. In the present study, betadine (10% povidone iodine; 1% free iodine) was used as scolicidal agent. Even though WHO (1996) (7) recommends hypertonic saline as scolicidal, due to the lack of easy availability of 20% hypertonic saline, it could not be used in the study. No sclerosing agent such as alcohol was used for destruction of the germinal layer unlike studies conducted by Ustunsoz et al. (1) and vanSonnenberg et al. (20). Betadine was chosen as the scolicidal agent, as it is easily available and has minimal side effects. Many investigators use various protocols for oral therapy (7, 19, 21). WHO (7) has suggested initiation of oral albendazole therapy at least four hours before the procedure. In our study, oral albendazole was prescribed for all patients 4 days before the procedure and continued for one month after the procedure. The therapy was continued for three months in cases who had significant residual cystic areas at one month follow-up sonography. In such cases, after three weeks of oral albendazole therapy, one week gap was given in order to prevent any hepatotoxic effects of continuous therapy. None of the patients developed derangement of the hepatic functions during the course of therapy. In a meta-analysis by Paksoy et al. (22) PAIR therapy along with oral albendazole was effective in the management of hepatic echinococcosis. Many long term results indicate that PAIR therapy is as efficacious as surgery and has a low complication rate in comparison (23, 24). It is the preferred modality of treatment in children (24). In this study, reduction in cyst size was observed in all 15 patients. Bosanac et al. (15) observed cyst size reduction in all patients of their study. Solidification was observed in 11 (73.3%) in the present study. Goktay et al. (24) observed solidification in about 83% of the cases. They used hypertonic saline as the scolicidal agent. Foci of calcification were seen in seven (46.7%) patients in our study. Residual cystic area remained in four (26.7%); however, this residual cystic area was observed at a short term follow-up period of 1year. This was probably due to the large size of the cysts and presence of multiple daughter cysts. Goktay et al. (24) did not observe any significant residual cystic areas over a follow-up period of three years. Complications such as anaphylaxis, peritoneal seedlings and secondary recurrence were not observed in the present study. Many authors (1, 15, 17, 21) did not observe these complications in their studies. Short hospital stay associated with minimal invasiveness of the procedure makes PAIR therapy a cost effective treatment option for hepatic hydatid cysts. From the present study, it is concluded that PAIR therapy using betadine as the scolicidal agent is one of the minimally invasive, effective and less expensive treatment options for appropriately selected cases of hydatid disease. It should be offered to all the patients with Gharbi type I-III hepatic hydatid cysts, patients who refuse surgery and have associated co-morbid conditions which contraindicate surgery.

## References

[1] Ustunsoz B, Akhan O, Kamiloglu MA, Somuncu I, Ugurel MS, Cetiner S (1999). Percutaneous treatment of hydatid cysts of the liver: long-term results.. AJR Am J Roentgenol..

[2] Nepalia S, Joshi A, Shende A, Sharma SS (2006). Management of echinococcosis.. J Assoc Physicians India..

[3] Aygun E, Sahin M, Odev K, Vatansev C, Aksoy F, Paksoy Y (2001). The management of liver hydatid cysts by percutaneous drainage.. Can J Surg..

[4] Schipper HG, Lameris JS, van Delden OM, Rauws EA, Kager PA (2002). Percutaneous evacuation (PEVAC) of multivesicular echinococcal cysts with or without cystobiliary fistulas which contain non-drainable material: first results of a modified PAIR method.. Gut..

[5] Smego RA, Jr, Sebanego P (2005). Treatment options for hepatic cystic echinococcosis.. Int J Infect Dis..

[6] Akhan O, Ozmen MN, Dincer A, Sayek I, Gocmen A (1996). Liver hydatid disease: long-term results of percutaneous treatment.. Radiology..

[7] Bulletin of WHO on PAIR therapy (1996). Department of Communicable Disease, Surveillance and Response..

[8] Nasseri Moghaddam S, Abrishami A, Malekzadeh R (2006). Percutaneous needle aspiration, injection, and reaspiration with or without benzimidazole coverage for uncomplicated hepatic hydatid cysts.. Cochrane Database Syst Rev..

[9] Mueller PR, Dawson SL, Ferrucci JT, Jr, Nardi GL (1985). Hepatic echinococcal cyst: successful percutaneous drainage.. Radiology..

[10] Khuroo MS, Zargar SA, Mahajan R (1991). Echinococcus granulosus cysts in the liver: management with percutaneous drainage.. Radiology..

[11] Polat KY, Balik AA, Oren D (2002). Percutaneous drainage of hydatid cyst of the liver: long-term results.. HPB (Oxford)..

[12] Sbihi Y, Rmiqui A, Rodriguez-Cabezas MN, Orduna A, Rodriguez-Torres A, Osuna A (2001). Comparative sensitivity of six serological tests and diagnostic value of ELISA using purified antigen in hydatidosis.. J Clin Lab Anal..

[13] Gharbi HA, Hassine W, Brauner MW, Dupuch K (1981). Ultrasound examination of the hydatic liver.. Radiology..

[14] Polat P, Kantarci M, Alper F, Suma S, Koruyucu MB, Okur A (2003). Hydatid disease from head to toe.. Radiographics..

[15] Bosanac ZB, Lisanin L (2000). Percutaneous drainage of hydatid cyst in the liver as a primary treatment: review of 52 consecutive cases with long-term follow-up.. Clin Radiol..

[16] Saremi F (1992). Percutaneous drainage of hydatid cysts: use of a new cutting device to avoid leakage.. AJR Am J Roentgenol..

[17] Saremi F, McNamara TO (1995). Hydatid cysts of the liver: long-term results of percutaneous treatment using a cutting instrument.. AJR Am J Roentgenol..

[18] Acunas B, Rozanes I, Celik L, Minareci O, Acunas G, Alper A (1992). Purely cystic hydatid disease of the liver: treatment with percutaneous aspiration and injection of hypertonic saline.. Radiology..

[19] Keshmiri M, Baharvahdat H, Fattahi SH, Davachi B, Dabiri RH, Baradaran H (2001). Albendazole versus placebo in treatment of echinococcosis.. Trans R Soc Trop Med Hyg..

[20] vanSonnenberg E, Wroblicka JT, D'Agostino HB, Mathieson JR, Casola G, O'Laoide R (1994). Symptomatic hepatic cysts: percutaneous drainage and sclerosis.. Radiology..

[21] Giorgio A, Tarantino L, de Stefano G, Francica G, Mariniello N, Farella N (2001). Hydatid liver cyst: an 11-year experience of treatment with percutaneous aspiration and ethanol injection.. J Ultrasound Med..

[22] Paksoy Y, Odev K, Sahin M, Arslan A, Koc O (2005). Percutaneous treatment of liver hydatid cysts: comparison of direct injection of albendazole and hypertonic saline solution.. AJR Am J Roentgenol..

[23] Etlik O, Arslan H, Bay A, Sakarya ME, Harman M, Temizoz O (2004). Abdominal hydatid disease: long-term results of percutaneous treatment.. Acta Radiol..

[24] Goktay AY, Secil M, Gulcu A, Hosgor M, Karaca I, Olguner M (2005). Percutaneous treatment of hydatid liver cysts in children as a primary treatment: long-term results.. J Vasc Interv Radiol..

